# Changes in Methylation across Structural and MicroRNA Genes Relevant for Progression and Metastasis in Colorectal Cancer

**DOI:** 10.3390/cancers13235951

**Published:** 2021-11-26

**Authors:** Nitin Patil, Mohammed L. Abba, Chan Zhou, Shujian Chang, Timo Gaiser, Jörg H. Leupold, Heike Allgayer

**Affiliations:** 1 Department of Experimental Surgery—Cancer Metastasis, Mannheim Medical Faculty, Ruprecht Karls University of Heidelberg, 68167 Mannheim, Germany; Nitin.Patil@medma.uni-heidelberg.de (N.P.); Mohammed.Abba@medma.uni-heidelberg.de (M.L.A.); chan.zhou@medma.uni-heidelberg.de (C.Z.); xdw@jiangnan.edu.cn (S.C.); joerg.leupold@medma.uni-heidelberg.de (J.H.L.); 2 Institute of Pathology, Mannheim Medical Faculty, Ruprecht Karls University of Heidelberg, Theodor Kutzer Ufer 1-3, 68167 Mannheim, Germany; Timo.Gaiser@umm.de

**Keywords:** genome-wide methylation array, colorectal cancer, methylation, miRNA, metastasis

## Abstract

**Simple Summary:**

Changes in the expression of key molecules such as microRNAs (miRs) can drive or suppress carcinogenesis and metastasis. A number of established transcriptional and genetic mechanisms regulate miR gene expression, but methylation/epigenetics have been analyzed less. Here, we systematically evaluated genome-wide methylation changes, focusing on miR, downstream targets, and further genes relevant for metastasis in colorectal cancers (CRC), including CpG islands, open seas, and north and south shore regions. A number of miRs deregulated during CRC progression/metastasis were significantly affected by methylation changes, especially within CpG islands and open seas. Several of these miRs cooperate in cancer- and metastasis-related pathways, while methylation changes otherwise primarily affect protein-coding genes. Our results highlight alternative routes to the transcriptional and genetic control of miR and further gene expression relevant for CRC progression and metastasis by changes in gene methylation. They also bear important therapeutic implications since drugs that alter methylation states are now in clinical use.

**Abstract:**

MiRs are important players in cancer and primarily genetic/transcriptional means of regulating their gene expression are known. However, epigenetic changes modify gene expression significantly. Here, we evaluated genome-wide methylation changes focusing on miR genes from primary CRC and corresponding normal tissues. Differentially methylated CpGs spanning CpG islands, open seas, and north and south shore regions were evaluated, with the largest number of changes observed within open seas and islands. Kyoto Encyclopedia of Genes and Genomes (KEGG) pathway enrichment analysis revealed several of these miRs to act in important cancer-related pathways, including phosphatidylinositol 3-kinase (PI3K)–protein kinase B (Akt) and mitogen-activated protein kinase (MAPK) pathways. We found 18 miR genes to be significantly differentially methylated, with MIR124-2, MIR124-3, MIR129-2, MIR137, MIR34B, MIR34C, MIR548G, MIR762, and MIR9-3 hypermethylated and MIR1204, MIR17, MIR17HG, MIR18A, MIR19A, MIR19B1, MIR20A, MIR548F5, and MIR548I4 hypomethylated in CRC tumor compared with normal tissue, most of these miRs having been shown to regulate steps of metastasis. Generally, methylation changes were distributed evenly across all chromosomes with predominance for chromosomes 1/2 and protein-coding genes. Interestingly, chromosomes abundantly affected by methylation changes globally were rarely affected by methylation changes within miR genes. Our findings support additional mechanisms of methylation changes affecting (miR) genes that orchestrate CRC progression and metastasis.

## 1. Introduction

CRC is presently the second leading cause of cancer deaths and third most commonly diagnosed cancer worldwide [1]. The mortality associated with CRC is largely due to its ability to establish distant metastases, with the 5-year survival rate for metastatic CRC being approximately 10% without treatment [2].

The successive acquisition of genetic and epigenetic alterations has been shown to drive the initiation and progression of adenomas to carcinomas in CRC. These mediate the transformation of a normal colorectal epithelium to a benign adenoma, and the accumulation of further multiple genetic and epigenetic changes in particular clones can result in an invasive and metastatic phenotype [3,4,5]. A multitude of research efforts have sought to identify and investigate the key molecules involved in the initiation and progression of CRC. A large number of molecular drivers have been identified, of which molecules such as adenomatous polyposis coli (APC), tumor protein P53 (TP53), kirsten rat sarcoma virus (KRAS), and catenin beta-1 (CTNNB1) appear to play crucial roles [4].

Almost three decades ago, a group of small non-coding RNAs was identified, which renders important mediators of post-transcriptional gene regulation. This group of molecules, also called miRs, represents small endogenous RNA molecules (18–22 nt) that repress the expression of protein-coding genes [6,7], the predominant function of miRs being RNA silencing and the negative regulation of gene expression at the post-transcriptional level [8]. The interaction of miR seed sequences with sequences in the 3′ untranslated region (UTR) of their target mRNAs leads to translational repression. Interestingly, miR binding sites have also been identified in other mRNA regions, including the 5′ UTR and coding sequence as well as within promoter regions [9,10]. The analyses of large patient datasets of diverse cancer entities identified over 10,000 miR–mRNA interactions to be associated with cancer progression. Almost 40% of these interactions exhibited a high fidelity of miR function [11]. The aberrational regulation of miRs has been shown to interfere with several important signaling cascades including epidermal growth factor receptor (EGFR), kirsten rat sarcoma virus (KRAS), PI3K, Wingless and Int-1 (Wnt), myelocytomatosis (Myc), HIPO and Notch pathways, amongst others, which are vital to tumor progression and metastasis [12]. In addition, an accumulating number of studies, including our own, make it clear that miRs are important players in different steps of metastasis in multiple cancer types, including CRC [5,13,14,15,16,17]. The means of regulation of miR gene expression in this context can be different; however, most studies so far have investigated, and demonstrated, changes in transcription as major mechanisms of regulating miR expression during metastasis [17,18].

Epigenetic modifications have emerged as a major mechanistic hallmark that drives malignant diseases, with the most prominent epigenetic changes comprising the methylation of CpG islands, the methylation of histone proteins, and deacetylation [19]. It is now well established that aberrant epigenetic modifications play a critical role in cancer progression and metastasis irrespective of genetic lesions [20]. Comparatively, malignant cells have been described to be typically hypermethylated at CpG islands [21].

In this study of colorectal carcinomas, we explored genome-wide methylation changes, specifically focusing on miRs genes due to the important role they play in cancer progression and metastasis. Toward this end, we selected all miR gene regions that were affected by methylation including the gene body, islands, shelves, shores, and open seas. KEGG pathway enrichment analysis revealed that the mRNAs these miRs regulate play important roles in cancer progression and metastasis. Using a two-fold (up or down) differential methylation difference between tumor and normal samples, we found 18 miRs to be differentially methylated in tumor samples, nine of them being hypomethylated and nine hypermethylated as compared to normal colorectal tissue. In line with the literature and own previous studies, these miRs, and their deregulated expression in CRC, have been identified to play potent roles in cancer progression and metastasis. Our findings support additional mechanisms orchestrating CRC progression and metastasis by affecting gene and miR regulation via methylation.

## 2. Material and Methods

### 2.1. Tissue Material and Ethical Consent

In general, all of the samples were analyzed completely anonymized, retrospectively, and without the possibility to track back any results to the individual patient. The study was approved by the local board of ethics (Medical Ethics Committee II, University of Heidelberg), ethics approval: 2012-608R-MA, to T.G. Information regarding UICC staging and pathological grading were collected in line with the stipulated international formats [22,23]. Tissue specimens from tumor and corresponding normal mucosa distant from the tumor site were collected after macroscopic verification by a pathologist, and frozen immediately in liquid nitrogen. In total, samples of 24 patients were analyzed in the study (24 tumor and 24 matched normal samples). All patients were of Caucasian descent.

### 2.2. Genomic DNA Isolation

Genomic DNA was isolated from resected tumor and corresponding normal samples using the QIAamp DNA mini kit (Qiagen GmbH, Hilden Germany) according to the manufacturer’s instructions. DNA concentration was measured with the Nanodrop spectrophotometer and 500–1000 ng of DNA/sample were used in later experiments.

### 2.3. Methylation Profiling

DNA samples were submitted to the Genomics and Proteomics core facility of the German Cancer Research Center (DKFZ), Heidelberg Germany for methylation profiling using the Illumina Infinium 450 K Methylation Array according to the standard protocol. In summary, DNA samples were bisulfite converted using the Zymo EZ DNA Methylation Kit. Then, the bisulfite converted DNA was denatured and further amplified. Afterwards, the DNA were fragmented using enzymatic digestion with FMS fragmentation solution and then precipitated. Then, the re-suspended DNA fragments were hybridized to the BeadChip. After an overnight incubation step, the un-hybridized probes were washed away, and the BeadChip was stained and scanned with the Illumina iScan system.

### 2.4. Bioinformatics Analysis

The level of methylation was determined at each locus by the intensity of the two possible fluorescent signals from the C (methylated) and T (unmethylated) alleles. Pre-processing was done in two steps, using the R package “minfi” [24]. Background subtraction was followed by normalizing to internal controls that were applied to the Meth and Unmeth intensities separately. Filtering was done according to Sturm et al. [25] by the removal of probes targeting the X and Y chromosomes, the removal of probes containing a single-nucleotide polymorphism (dbSNP132 Common) within five base pairs, by including the targeted CpG site, and probes not mapping uniquely to the human reference genome (hg19), allowing for one mismatch. In total, 438,370 probes were subjected to analysis. For analysis, the relative level of methylation was calculated as the ratio of the methylated probe signal to total locus signal intensity (beta value).

Pairwise comparisons were performed using the Wilcoxon signed-rank test. For multiple testing, the step-down maxT testing procedure was applied to provide strong control of the family-wise type I error rate [26]. Genome annotation was based on the University of California Santa Cruz (UCSC) Genome Browser (http://genome.ucsc.edu, accessed on 20 September 2021; UCSC Genome Bioinformatics, Santa Cruz, CA, USA), whereas miR annotation was from miRBase (http://www.mirbase.org, accessed on 20 September 2021) Release 22.

### 2.5. Data Availability

All methylation data discussed in the manuscript have been deposited in the NCBI Gene Expression Omnibus and can be accessed using the GEO Series accession number GSE184494 (https://www.ncbi.nlm.nih.gov/geo/query/acc.cgi?acc=GSE184494, accessed on 20 September 2021).

### 2.6. KEGG Analysis

All potential mRNA targets of all miR genes were individually identified using the Targetscan and miRDB online tools. Then, the common gene signatures of the individual miRs were imported into the DAVID online tool (david.abcc.ncifcrf.gov, accessed on 29 September 2021), and all functional KEGG pathways were identified [27]. All significant pathways were considered (*p* < 0.05), and the most frequently delineated pathways were used in the final analysis. The generated pathways that were irrelevant to cancer in general or CRC specifically were manually curated. Then, the resulting list of pathways was used to generate a heat map in Microsoft Excel based on the frequency of occurrence of the given miRs. Furthermore, canonical pathways that interacted with the highest number of miRs as well as miRs that individually interacted with the most pathways were delineated.

### 2.7. Ingenuity Pathway Analysis (IPA)

The IPA pathway tool from QIAGEN Germany was used for the analysis. All predicted targets of mature miRs encoded by hyper and hypomethylated mRNA genes were imported into the ingenuity pathway core analysis pipeline using the default settings with the exception of following changes. Node types were limited to canonical pathways, disease, function, fusion gene product, G-protein coupled receptor, mature miR, miR, and others. Species was limited only to humans. Tissues and cell filters were limited to cancer or colorectal disease. The mutation filter was set to functional effect and translational impact. From the resulting pathways, only the top hit pathways with oncogenic relevance were selected.

## 3. Results

### 3.1. Methylation Array and Associated Bioinformatics: General Distribution of Differentially Methylated Sites between Coding, Non-Coding, and Intergenic Regions

Tissue samples from 24 matched primary CRC and corresponding normal colorectal tissue pairs were profiled on the Infinium 450 K Bead Array. The analyzed samples were completely anonymized, without the possibility to track back any results to the individual patient. The median age of the patients at diagnosis was 65 years; 38% were females, and 62% were males. There was no evidence of a familiar hereditary background in all cases. Only 4% of the patients had pT1 stages, while 21% had pT2, 58% had pT3, and 17% had pT4 stages, this being comparable to the distribution of stages within other, also larger western CRC cohorts [1,28]. Half (50%) of the patients had pN0 and 50% had pN1-2 stage. As far as clinical information was available, five patients showed clinically diagnosed metastasis (M1) to the liver (Supplementary Table S1).

The output of differentially methylated genes between colorectal tumor and corresponding normal tissues comprised both protein and non-protein coding genes. For each methylated CpG site, the median tumor and normal beta values, together with *p*-values, were analyzed using the Wilcoxon Signed-Rank test. For all genes that were significantly differentially methylated (*p* ≤ 0.05), the median difference beta values were calculated to determine if the genes were hyper- or hypomethylated with respect to the normal colorectal samples. The methylated sites for the genes were also mapped to correspond to CpG islands, north and south shores, north and south shelves, as well as open seas. We took the CpG island definition of a 200 bp region of DNA with a GC content higher than 50% and an observed CpG versus expected CpG ratio greater or equal to 0.6. We considered methylation sites up to 2 kb upstream/downstream of CpG islands as north and south shores, respectively, and shelves as −4 kb upstream/downstream of CpG islands. Open seas represented isolated CpGs within the genome >4 kb from CGIs. Moreover, the transcriptional start site (TSS) TSS200 and TSS1500 regions represent sites that are located up to −200 and −1500 bp upstream of the transcriptional start site, respectively. The definitions of north and south shores, north and south shelves, and open seas were applied as previously published [29].

**Figure 1:**
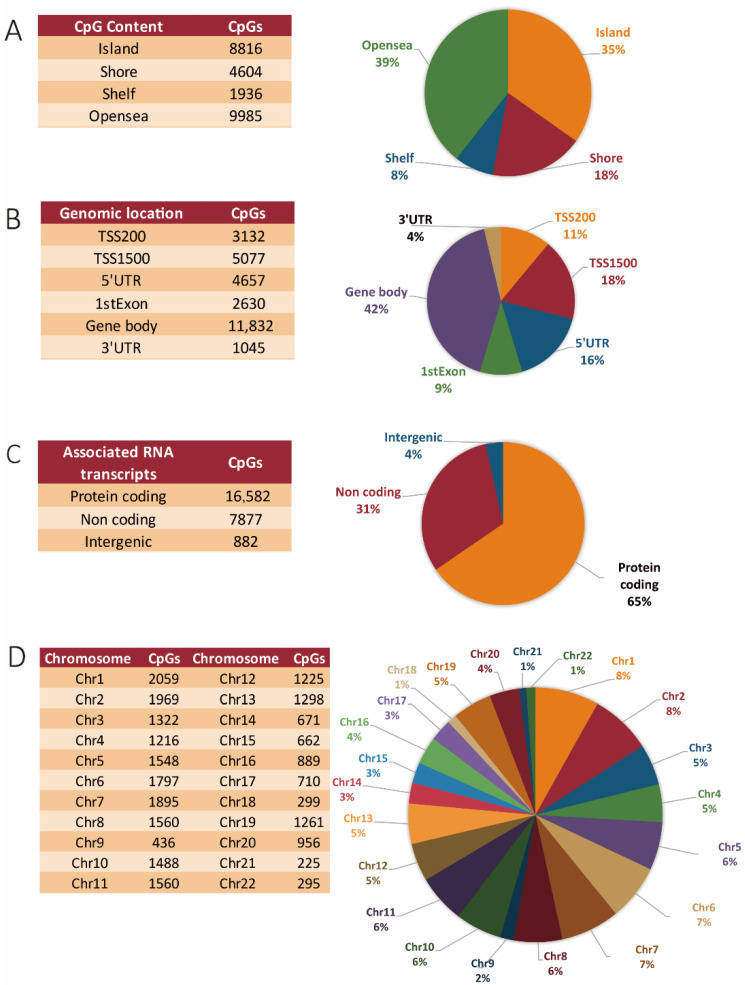
DNA methylation landscape in CRC tissues. (**A**) Genomic distribution of CpG sites in relation to CpG islands and neighboring shores, shelves, and open seas. (**B**) Functional genomic and neighborhood location and distribution of methylated CpG sites. (**C**) Distribution of CpGs in relation to coding, non-coding, and intergenic regions, respectively. (**D**) Chromosome distribution of the differential methylated sites.

Globally, 34.8% of differentially methylated CpGs occurred in islands, 18.2% occurred in shores, 7.6% occurred in shelves, and 39.4% occurred in open seas (Figure 1A). Specifically, 18% of the methylated regions were found within the TSS1500, 11% were found in the TSS200 site, respectively; 16% were found within 5′ UTRs, 9% were found within the 1st exons of genes, 42% were found in the gene body, and 4% were found in the 3′ UTR regions, respectively (Figure 1B). Regions neighboring the gene body at both the 5′ and 3′ flanking regions also showed significant methylation differences, with 5′ UTRs accounting for 4657 and the 3′ UTRs with 1045 differentially methylated sites, respectively (Figure 1B). More than half (65%) of the aberrant DNA methylation sites were associated with protein-coding genes, 31% were associated with genes for non-coding RNAs (including miR genes, long non-coding RNAs (lncRNA) genes, etc.), and 4% were located in intergenic regions (Figure 1C).

As shown in Figure 1D, the overall changes in DNA methylation were mainly seen within chromosome 1 (8.13%), followed by chromosomes 2 (7.77%) and chromosome 7 (7.48%). The least affected chromosomes were chromosome 9 (1.7%), chromosome 18 and 22 (both 1.1%), and chromosome 21 (0.88%). The global methylation pattern across all chromosomes, including the proportions of both hyper- and hypomethylated CpGs in tumor versus corresponding normal tissue, and their relations to the specific genomic features (islands, shelves, shores, and open seas) is represented in Table 1. Our global analysis shows that methylation changes predominantly affected protein-coding genes. Methylation preferentially occurred within gene bodies, and the chromosomal distribution was relatively proportional to chromosome size, with a few exceptions. The majority of methylation changes were seen on chromosomes 1, 2, 7, 6, 11, 8, 5, 10, 3, 13, 19, 12, and 4, respectively, in decreasing order of magnitude. The other autosomes were less affected, with chromosomes 21, 22, 18, and 9 showing the least changes in their methylation pattern in tumor as compared to normal tissue (Figure 1D).

### 3.2. Hypermethylated and Hypomethylated CpG Areas across Genomic Features

**Figure 2:**
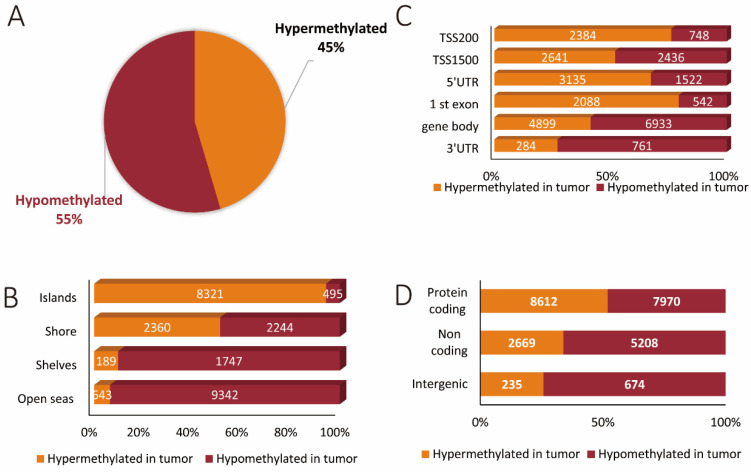
The methylation landscape of all genes across genomic features relative to hypermethylated and hypomethylated states. (**A**) Relative contribution of unique hypermethylated and hypomethylated CpG sites. (**B**) Percentages of CpG hypermethylation and hypomethylation events according to their CpG content and neighborhood context. (**C**) The distribution of hypermethylated and hypomethylated CpGs examined in different functional genomic regions. (**D**) The differentially methylated sites within protein-coding genes, non-coding genes, and intergenic regions.

Next, we evaluated the location of all of the differentially methylated CpGs in relation to functional genomic regions and genomic features. Altogether, 11,513 (45%) sites were hypermethylated and 13,828 (55%) sites were hypomethylated in the tumor as compared to normal colorectal tissue (Figure 2A). Moreover, we found that open seas harbored the largest number of differentially methylated sites, with the greater majority of sites being hypomethylated (9342 sites) as opposed to only 643 being hypermethylated in the tumor as compared to corresponding normal tissues. The next most abundantly affected genomic feature was CpG islands comprising 8816 sites, of which the majority was hypermethylated (8321 as compared to 495 hypomethylated sites) in tumor tissue. In the case of shelves, we also observed a predominant hypomethylation of CpGs (1747) as compared to 189 hypermethylated sites in the tumor tissues. No significant difference in number was observed between hypermethylated (2360) and hypomethylated (2244) sites in shores (Figure 2B).

In a separate analysis, functional genomic regions were evaluated with a total of 28,373 differentially methylated sites. Here, methylation changes within the gene body were the most abundant (11,832 affected sites). Of these, 4899 were hypermethylated and 6933 hypomethylated in tumor as compared to normal colorectal tissues. Promoter regions within 200 and up to 1500 bp relative to the transcriptional start sites were the next most abundantly differentially methylated regions with 8209 sites. Of these, 5025 sites were hypermethylated and 3184 sites hypomethylated in tumor as compared to corresponding normal tissues (Figure 2C). These findings are in line with the overall observation that most of the methylation changes were observed in protein-coding regions (16,582 methylation sites) as opposed to 7877 sites for non-coding regions (Figure 2D).

### 3.3. Methylation-Specific Patterns across miR Genes

The methylation pattern in miR genes across all chromosomes, including the contribution of both hypermethylated and hypomethylated CpGs, and their context to specific genomic features (islands, shelves shores, and open seas) is represented in Table 2. Interestingly, for miR genes, methylation changes occurred predominantly in promoter regions located within 1500 bp from transcription start sites. In addition, chromosomes 14, 20, and 19 accounted for over 43% of the methylation changes observed for miR genes in tumor versus normal tissue. An interesting observation was that chromosomes that were abundantly affected by methylation changes within the global gene profile were largely unaffected by methylation changes within miR genes. Of note, the chromosomes 4, 12, 17, 18, and 21 showed no methylation changes within miR genes (Table 2).

**Figure 3:**
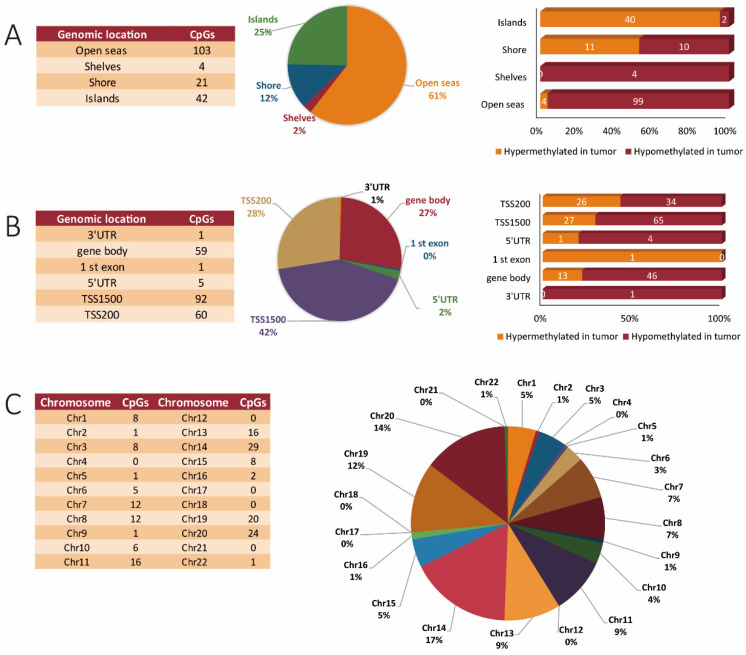
MiR gene methylation profiling in CRC tissues. (**A**) Genomic distribution of differentially methylated CpG sites in miR genes in relation to CpG islands, shores, shelves, and open sea regions. (**B**) The number and percentage of hypermethylated and hypomethylated CpG sites of miR genes ordered according to their functional genomic distribution. (**C**) Chromosome location of the differentially methylated sites of miR genes.

Overall, similar trends were observed for the miR genes as with the whole genome profile. When we specifically focused on miR genes, we found 170 unique sites that were differentially methylated, with 115 being hypomethylated and 55 being hypermethylated in the tumors. Regarding their location within functional genomic regions, the highest number of differentially methylated sites was observed in open seas (103 sites), of which 99 were hypomethylated and only four were hypermethylated in tumor tissues. Overall, in the case of CpG islands, 42 differentially methylated sites were identified from which a total of 40 sites were hypermethylated and only two were hypomethylated in tumor tissues when compared to corresponding normal tissues. Within shores, a total of 21 differentially methylated sites were identified of which 10 sites were hypomethylated and 11 were hypermethylated in tumor as compared to normal tissue. Only four sites were hypomethylated within sea shelves; none were found here that were hypermethylated (Figure 3A). A more focused evaluation of the miR genes interestingly showed promoter regions to be more abundantly hit by methylation changes as opposed to the gene body, as shown in Figure 3B. The 5′ and 3′ UTR regions were minimally affected by methylation changes in the miR genes (Figure 3B). Interestingly, aberrantly methylated miR sites were found only in 17 autosomal chromosomes. Chromosomes 4, 12, 17, 18, 21, and sex chromosomes were devoid of miR methylation sites (Figure 3C).

### 3.4. Identification of Significant Differentially Methylated miRs

**Figure 4:**
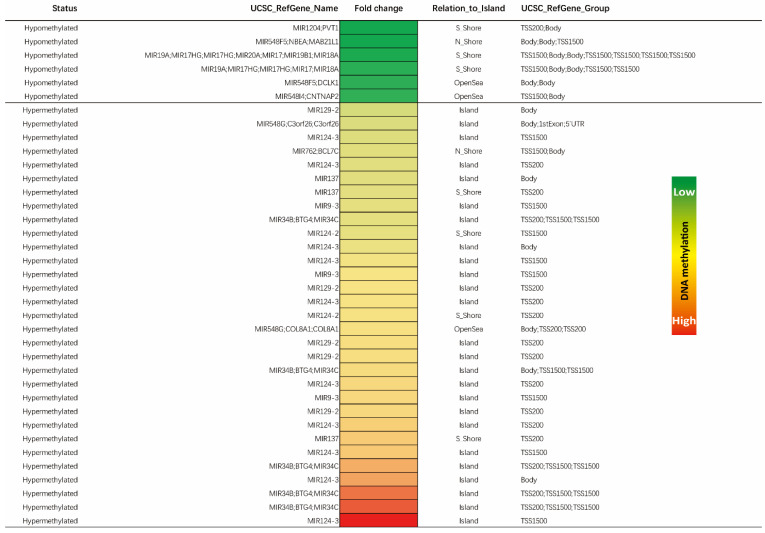
Heat map of differentially methylated miR genes. The heat map showcases all >2-fold differentially methylated CpG sites within miR genes between tumor and normal colorectal samples. A total of 37 unique sites covering 18 miR genes were identified (nine hypermethylated and nine hypomethylated). The color in each small box represents the relative methylation level of the individual positions within genes in colorectal carcinomas as compared to normal colorectal tissue. The light green color to red color represents a low to high relative methylation status of the individual site, respectively. For the sake of completeness, genes with overlapping open reading frames sharing similar genomic locations are also shown.

In total, 170 unique CpG sites were affected by methylation changes across miR genes. These sites concerned 107 distinct miR genes. In order to ascertain the most significant differentially methylated candidates, we selected all miR genes with a fold change methylation difference between tumor and normal tissue of two and above (hypomethylated and hypermethylated). This analysis revealed 37 distinct CpG sites within miR genes to be significantly hit by methylation changes. These sites represented 18 unique miR genes (Figure 4). Of these miR genes, MIR124-3 was the most hypermethylated in the tumor samples (16-fold methylation difference). The MIR1204 gene was the most hypomethylated in tumor samples when compared with corresponding normal resected tissues (−2.9-fold methylation difference). The regions responsible for these methylation changes were most visible within CpG islands and mostly affected TSS regions up to 1500 bp, as described in Figure 3B. These 18 unique miR genes were further analyzed for their reported impact on colorectal carcinogenesis and tumor progression.

Toward this end, we compared these microRNA genes that were affected by methylation changes with recent literature (Table 3) to see whether the observed methylation pattern correlated with current miR gene expression and functional data. In line with our findings, we saw most of our hypermethylated miRs described as downregulated in several tumor types including CRC, and to play a role as potential tumor and/or metastasis suppressors. Similarly, in case of hypomethylated miR genes found in this study, most studies supported an oncogenic and/or pro-metastatic role while being more highly expressed in diverse solid cancers, including CRC.

### 3.5. Pathway Enrichment Analysis of Differentially Methylated miR Genes

**Figure 5:**
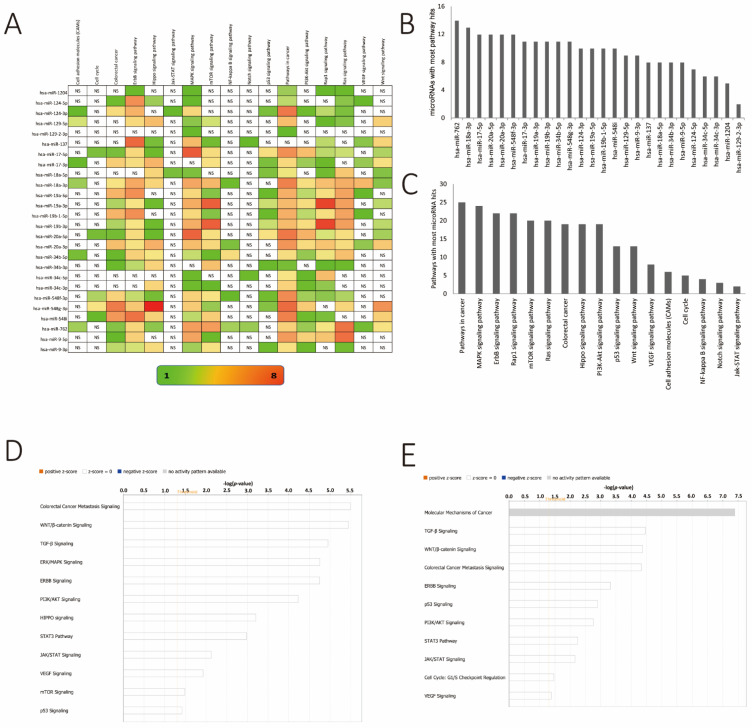
KEGG pathway enrichment analysis by using DAVID Ease. (**A**) Number of genes related to the enriched KEGG pathway. The color of the bar corresponds to log10 (*p*-value). (**B**) Bar diagram showcasing miRs regulating the highest number of canonical pathways in descending order. (**C**) Bar diagram showcasing canonical pathways regulated by the largest number of miRs from the differential methylation list (see Figure 4) in descending order. (**D**) Ingenuity pathway analysis of targets of hypermethylated miR genes. The threshold line is equivalent to a –log *p* value of 0.05. (**E**) Ingenuity pathway analysis of targets of hypomethylated miR genes. The threshold line is equivalent to a –log *p* value of 0.05.

To further explore the potential functions of differentially methylated sites affecting miRs, we identified all putative targets of the selected 18 miR genes, using the Targetscan and miRDB online tools. All miR targets were individually uploaded into the DAVID Ease online platform. Next, we carried out the KEGG enrichment analysis by using the DAVID bioinformatics resource. Here, we performed a functional and pathway enrichment analysis to map the differentially methylated genes to various types of molecular networks. All pathways with *p*-values < 0.05 for individual miRs were compiled and only pathways known to have a published impact on cancer development were considered. The lists of the pathways for each miR were analyzed together, and pathways that were common to all miRs were followed further. In total, 17 cancer-relevant pathways were identified for all of these miRs differentially methylated in their genes in our study, including the MAPK, EGFR, ras-proximate-1 or ras-related protein 1 (Rap1), mammalian target of rapamycin (mTOR), and Ras signaling pathway, the Hippo signaling pathway, the PI3K–Akt signaling pathways, and an implication of events leading to chromosomal instability (CIN) and microsatellite instability (MSI). The latter comprise several cellular pathways leading to the development of CRC (Figure 5A). The most recurrent pathways for all miRs implicated to be differentially methylated in CRC in our study were selected for further evaluation and are summarized in Figure 5. Additionally, we performed two further independent analyses, as shown in Figure 5B,C, respectively, to evaluate which of the individual pathways were targeted by the majority of the miRs we identified to be differentially methylated in their genes and vice versa. Our analysis showed that hsa-miR-762, hsa-miR-18a-3p, hsa-miR-17-5p, hsa-miR-20a-5p, hsa-miR-20a-3p, hsa-miR-548f-3p, hsa-miR-17-3p, hsa-miR-19a-3p, hsa-miR-19b-3p, hsa-miR-34b-5p, and hsa-miR-548g-3p targeted eleven or more of the pathways identified in the network (Figure 5B). All of these miRs have been implicated previously, by us and others, to be critical molecular players in the regulation of metastasis and/or CRC progression [54,55,58,71,72].

Furthermore, we evaluated the most recurring pathways targeted by all of these miRs together. Toward this end, the MAPK, ErbB, Rap1, mTOR, Ras signaling pathways as well as the Hippo and PI3K–Akt signaling pathways were regulated by all of these miRs, implicating changes in the methylation of the corresponding miR genes as a crucial event in the mediation of CRC progression and metastasis (Figure 5C). The identification of this network of important signaling pathways as targets of the differentially methylated miRs shown here further validates the vital role that these miRs play in CRC. To corroborate the DAVID analysis, pathways specific for gene targets of hypermethylated and hypomethylated miRs were evaluated using the IPA tool. For the hypermethylated miR targets, CRC metastasis signaling, WNT/β-catenin, and TGF-β signaling were the most significant pathways targeted (Figure 5D). For the hypomethylated miRs, molecular mechanisms of cancer, TGF-β signaling, and WNT/β-catenin were most visible as the targeted pathway groups in this tool (Figure 5E). Taken together, the pathway analyses from DAVID Ease and IPA analysis had very similar outcomes.

## 4. Discussion

The significant role played by miRs in the mediation of colorectal carcinogenesis, progression, and metastasis is well established. Depending on the genes targeted, miRs could have both oncogenic or tumor-suppressor functions. This is evident from several studies in CRC as well as in other solid carcinoma [14,15,17,36,39,58,60,71,73]. Importantly, the expression of any miR is only one factor to define its netto influence on its target genes; other parameters are, e.g., the specificity of interaction of the miR with its target mRNA (seed) sequence, the accessibility of the target mRNA for the microRNA by, for example, intracellular compartmentalization, and others [7,9,74]. The abundance of miR expression is being regulated at a number of levels, of which especially genetically acting ones have been abundantly studied so far, especially the transcriptional regulation of gene expression but also copy number changes; however, of course, epigenetics and especially the methylation of CpG sites could play a role as in other genes as well [5,8,19]. Interestingly, very few studies have investigated the impact of methylation on global miR gene expression so far. Moreover, most of these studies have been limited to studying methylation changes within the gene promoters only [29,75,76].

However, in our present study, we performed a genome-wide methylation analysis, covering 99% of all RefSeq genes and also comprising low CpG island density, which could remain undetected using other capture methods [29,77]. Furthermore, our evaluation of methylation changes was not only limited to promoter regions but also included shelves, shores, and open sea regions. A total of 25,341 CpG sites were found to be differentially methylated between colorectal tumor and corresponding normal samples (*p* < 0.05, Wilcoxon Signed-Rank test). Furthermore, most of the changes were observed within CpG island (35%) and open seas (39%). Interestingly, a greater proportion of differentially methylated sites were found in the gene body, which was followed by promoter regions up to 1500 base pairs from transcriptional start sites. Expectedly, most methylation changes occurred in the promoter region of coding genes. These findings are in line with published data that the methylation of promoters, especially those of tumor-suppressor genes, leads to a disruption of functional protein expression, which plays a critical role in cancer progression and other important functional processes [78,79].

The distribution of methylation changes across the different chromosomes was relatively proportional to chromosome size, with chromosomes 1, 2, 7, and 6 accounting for the most changes. These findings mirror those of other studies that have investigated chromosome and genome-wide methylation profiles [80,81]. Although both hypermethylation and hypomethylation events are common in cancer, the literature indicates that hypomethylation events are slightly more common [82]. Similarly, in our study, we also found both hypermethylation and hypomethylation events in the tumor as compared to normal tissues, with hypomethylation being slightly more predominant, with 55% hypomethylated as compared to 45% hypermethylated sites globally. Interestingly, hypomethylation and hypermethylation events were not evenly spread across genomic features or regions. Over 90% of the hypermethylation changes observed in tumor tissues were located within CpG islands, whereas the exact opposite was evident in open seas. Across all genomic regions, hypomethylation was more predominant with the exception of 3′ UTR and 1st exon regions.

Interestingly, hypermethylation was more evident in protein-coding genes, but the reverse was the case in non-coding regions, leading to the hypothesis that epigenetic alterations in coding and non-coding sequences might cooperate in human tumorigenesis. In line with the objectives of our study, we proceeded to specifically evaluate miR genes, many of which have been shown to regulate CRC progression and metastasis [14,36,37,40,42,43,54,60,65,66,68,69,83]. For miR genes, differential methylation was observed predominantly in the open seas and CpG islands. Functionally, these methylation events were more visible in the regions of −200 to 1500 base pairs relative to the transcription start sites. As mentioned above, over 95% of methylation events in open seas were hypomethylated, and those in CpG islands were hypermethylated. Surprisingly, the abundantly methylated chromosomes seen globally were not the same for the miR genes, with chromosomes 14, 20, 19, 13, and 11 mostly hit by methylation events within miR genes in contrast to the overall methylation pattern. A previous compilation by Ghorai and Ghosh identified chromosomes 1, 14, and 19 to harbor the largest number of cancer-associated miRs in the human genome [84]. These chromosomes with the highest number of miR genes overlap with the chromosomes hit by miR-gene specific methylation changes in our study. This underscores an implication that methylation is a potential key event in regulating tumor-associated miR expression, in addition to further mechanisms already shown to be essential, such as the transcriptional regulation of miRs, copy number changes, or changes in subcellular localization such as cytosolic, nuclear, or a concentration in exosomes to guide miR activity toward certain compartments in cancer [5,9,14,18,82]. To further focus on the most significant differentially methylated miR genes, we chose all sites that showed at least a two-fold methylation difference between tumor and corresponding normal samples. Using this selection criterion, we found 37 unique miR-related CpG sites. The majority of these significant differentially methylated sites were located in CpG islands, most of these occurring within 1500 base pairs from the transcriptional start site. The 37 unique CpG sites were located within a total of 18 distinct miR genes. Of these 18 miRs genes, nine genes were hypomethylated and nine genes were hypermethylated. Many of these miRs we found significantly differentially methylated in CRC also already have been shown to take functional roles in diverse further cancer entities besides CRC, which were in part related to changes in the methylation of their genes. For example, a tumor-suppressor function has been ascribed to all three loci encoding mature hsa-miR-124 (hsa-miR-124-1/-2/-3), which has been shown to be hypermethylated in cervical tumors [30], prostate cancer [31], nasopharyngeal carcinoma [85], HCC [32,33], bladder cancer [34], and CRC [35,36,37]. Likewise, mature miR-129-2 miR has been shown to be a tumor suppressor in esophageal cancer [38], breast cancer [39], and CRC [40]. The hypermethylation of miR-137 was observed in endometrial cancer [41], CRC [42,43], and pancreatic cancer [44,45].

MiR miR-34 family members have been well described as tumor suppressors by our own group in CRC [71] and by others in different kinds of carcinomas including cervical cancer [46], lung adenocarcinoma [47,48], breast cancer [49], oropharyngeal cancer [50], NSCLC [51], nasopharyngeal carcinoma [52], and prostate cancer [53]. Moreover, Toyota et al. demonstrated the downregulation of miR-34b/c expression in a panel of colorectal tumor tissues, again confirming these miRs as tumor suppressors in CRC [54]. These results also mirror our own findings. Other hypermethylated miR genes in our present study include miR-548G and the miR-9 family, which comprises tumor-suppressor miRs seen in Hodgkin’s lymphoma [56] and gastric cancer [57] so far.

Significantly hypomethylated miR genes in our present study included miR-1204 with established roles in breast cancer [58] and glioblastoma [59]. The mir-17-92 polycistron encodes six individual miR transcripts comprising miR-17, 18a, 19a, 20, 19b, and 92a. From our analysis, several members of this polycistron were hypomethylated, including MIR17HG, a known promoter of tumorigenesis and metastasis in CRC [60], miR-18a (miR-18a) having been shown to be important in prostate cancer [61], breast cancers [62], osteosarcoma [63], and nasopharyngeal carcinoma [64]. Additionally, miR-19a with documented roles to promote proliferation and migration in CRC [65,66], gastric cancer [67], and HCC [68] was hypomethylated in our analysis. Likewise, miR-20a has been shown to have a tumor-promoting activity in CRC [69]. Taken together, it is an established notion that the miRs we found as significantly changed in the methylation of their genes in colorectal carcinomas, as opposed to normal tissues, are highly relevant molecules that contribute to diverse aspects of (CRC) carcinogenesis, tumor progression, and metastasis.

This is further underlined by our evaluation of the canonical pathways that were attributable to the mRNA molecular targets of these particular miRs. We discovered that several of the miRs might act as a network regulating essential (CRC) cancer-associated pathways, e.g., the EGFR, MAPK, Ras, or the mTOR signaling pathway, amongst others. These strongly enriched canonical pathways, in addition to others, contributed to a generalized and significant enrichment of pathways in cancer in our study. Our findings are supported by the work of Sanchez-Vega et al. who, using 9125 samples from 33 cancer types, found similar pathways to be equally important. Interestingly, this study included DNA methylation changes in addition to mutations, copy number changes, mRNA expression, and gene fusion data to decode these pathway signatures [12]. With our own studies, using our data from miR expression analysis and whole genome sequencing, we were also able to postulate important contributions from a number of these differentially methylated miRs to CRC metastasis as a result of alterations in the pathways mentioned above [5,13,14].

Certainly, our study does have limitations, some of them being the small sample size and the non-availability of corresponding expression data from the same patients. Moreover, a sample triplet comprising tumor, normal, and metastatic tissues from the same patients would have provided a more significant inference in the context of cancer progression and metastasis; however, as the scientific community is well aware, metastasis samples are extremely rare to receive for any experimental analysis. In addition, due to the completely anonymized design of our study we, unfortunately, are unable to associate particular methylation changes to specific tumor stages of the cohort. Still, due to the fact that we studied a mixed population containing patients with some early, but, as a majority of cases, late cancer stages (e.g., 75% pT3 and pT4 stages, 50% pN1/2 stages, 5% M1), it is more likely that the methylation signature we identified is more representative of advanced tumor stages, possibly including features that support metastasis. Along these lines, we consider it interesting to speculate that the priming of certain genes/pathways that initiate or promote progression and/or metastatic steps by changes in methylation might already be visible in primary tumor samples.

Taken together, our present study, which is one of the few to perform genome-wide methylation analysis with a focus on microRNA genes, covering 99% of all RefSeq genes and also comprising low CpG island density, suggests it to be very likely that, besides other means of (genetic) deregulation, changes in methylation already at the primary tumor stage might contribute to the deregulation of expression of miRs and other (associated) genes, which contribute to advanced stages and metastasis development in CRC. Certainly, a definitive causal impact of our observed methylation changes can only be established with further experimental studies.

## 5. Conclusions

Taken together, our comprehensive analysis of differential miR gene methylation strongly implicates DNA methylation to have an important role in the regulation of a number of important miRs that regulate key cancer pathways in CRC, its progression, and its metastasis. It is interesting to speculate that methylation might have not only an equally important function in regulating miR gene expression in this and other cancer entities as compared to other means of regulation, such as transcription, mutations, or other genetic alterations, but that it might be more powerful by superimposing itself to modulate suchlike other means epigenetically. As a result, the modulation of methylation using clinically available therapeutic agents might be able to modulate essential miR-regulated molecular networks in CRC, and particular methylation events could be studied further as biomarkers in the risk classification of CRC.

## Figures and Tables

**Table 1:** Overview of genome-wide methylation burden across all chromosomes. The number of hypermethylation and hypomethylation events in relation to CpG islands and neighborhood regions are given.

Chromosome (Chr)	Status	OpenSea	Island	Shelf	Shore
Chr1	Hypomethylated	786	39	142	179
	Hypermethylated	57	636	10	210
Chr2	Hypomethylated	861	40	137	168
	Hypermethylated	36	568	5	154
Chr3	Hypomethylated	532	17	73	70
	Hypermethylated	30	430	15	155
Chr4	Hypomethylated	442	5	83	85
	Hypermethylated	12	462	7	120
Chr5	Hypomethylated	588	28	91	118
	Hypermethylated	62	541	4	116
Chr6	Hypomethylated	781	13	96	111
	Hypermethylated	75	503	17	201
Chr7	Hypomethylated	728	55	151	195
	Hypermethylated	50	533	21	162
Chr8	Hypomethylated	597	29	93	142
	Hypermethylated	26	502	7	164
Chr9	Hypomethylated	106	13	26	69
	Hypermethylated	12	177	5	28
Chr10	Hypomethylated	506	26	101	121
	Hypermethylated	40	573	5	116
Chr11	Hypomethylated	686	19	84	131
	Hypermethylated	41	448	4	147
Chr12	Hypomethylated	485	18	78	115
	Hypermethylated	43	357	20	109
Chr13	Hypomethylated	517	17	85	97
	Hypermethylated	29	372	11	170
Chr14	Hypomethylated	253	9	39	58
	Hypermethylated	13	220	5	74
Chr15	Hypomethylated	313	9	35	39
	Hypermethylated	9	219	4	34
Chr16	Hypomethylated	267	29	68	121
	Hypermethylated	35	295	15	59
Chr17	Hypomethylated	242	7	75	80
	Hypermethylated	27	229	4	46
Chr18	Hypomethylated	37	14	33	27
	Hypermethylated	1	161	4	22
Chr19	Hypomethylated	262	40	114	119
	Hypermethylated	37	524	18	147
Chr20	Hypomethylated	223	41	88	127
	Hypermethylated	2	385	5	85
Chr21	Hypomethylated	67	4	18	23
	Hypermethylated	5	88	0	20
Chr22	Hypomethylated	63	23	37	49
	Hypermethylated	1	98	3	21

**Table 2:** Overview of miR-specific methylation changes across all chromosomes. The number of CpG hypermethylation and hypomethylation events in tumor as compared to normal tissue is shown, according to their neighborhood context.

Chromosome	Status	OpenSea	Island	Shelf	Shore
Chr1	Hypomethylated	5	0	0	0
	Hypermethylated	0	1	0	2
Chr2	Hypomethylated	1	0	0	0
	Hypermethylated	0	0	0	0
Chr3	Hypomethylated	6	0	0	0
	Hypermethylated	1	1	0	0
Chr4	Hypomethylated	0	0	0	0
	Hypermethylated	0	0	0	0
Chr5	Hypomethylated	1	0	0	0
	Hypermethylated	0	0	0	0
Chr6	Hypomethylated	5	0	0	0
	Hypermethylated	0	0	0	0
Chr7	Hypomethylated	12	0	0	0
	Hypermethylated	0	0	0	0
Chr8	Hypomethylated	1	0	0	1
	Hypermethylated	0	3	0	7
Chr9	Hypomethylated	1	0	0	0
	Hypermethylated	0	0	0	0
Chr10	Hypomethylated	0	1	3	2
	Hypermethylated	0	0	0	0
Chr11	Hypomethylated	2	0	0	0
	Hypermethylated	2	12	0	0
Chr12	Hypomethylated	0	0	0	0
	Hypermethylated	0	0	0	0
Chr13	Hypomethylated	12	0	3	1
	Hypermethylated	0	0	0	0
Chr14	Hypomethylated	25	1	1	2
	Hypermethylated	0	0	0	0
Chr15	Hypomethylated	1	1	0	1
	Hypermethylated	0	5	0	0
Chr16	Hypomethylated	1	0	0	0
	Hypermethylated	0	0	0	1
Chr17	Hypomethylated	0	0	0	0
	Hypermethylated	0	0	0	0
	Hypomethylated	0	0	0	0
Chr18	Hypermethylated	0	0		0
	Hypomethylated	20	0	0	0
Chr19	Hypermethylated	0	0	0	0
	Hypomethylated	5	0	0	0
Chr20	Hypermethylated	0	18	0	1
	Hypomethylated	0	0	0	0
Chr21	Hypermethylated	0	0	0	0
	Hypomethylated	0	0	0	0
Chr22	Hypermethylated	1	0	0	0

**Table 3:** Methylation status of 16 of the miR genes found in this this study and supporting expression and functional data from the current literature on the respective miRs. For two miRs found in this study, supporting literature is not yet available to the best of our knowledge.

Hypermethylated miRs Found in Our Study	miR Expression in Cancer	Regulation	Role	Cancer Types	Target Genes	References
hsa-miR-124-2	Downregulated	Hypermethylated	Tumor suppressor	Cervical cancer	IGFBP7	[30]
hsa-miR-124-3	Downregulated	Hypermethylated	Tumor suppressor	Prostate cancer, Cervical cancer, HCC, Bladder cancer, CRC	IGFBP7, CRKL, Sp1, EDNRB, CCL20, DNMT3B, STAT3	[30,31,32,33,34,35,36,37]
hsa-miR-129-2	Downregulated	Hypermethylated	Tumor suppressor	Esophageal carcinoma, Breast cancer, CRC	SOX4, BCL2L2, BCL2	[38,39,40]
hsa-miR-137	Downregulated	Hypermethylated	Tumor suppressor	Endometrial cancer, CRC, Pancreatic cancer	EZH2, LSD1, TCF4, LSD1, KLF12, KDM4A	[14,41,42,43,44,45]
hsa-miR-34B	Downregulated	Hypermethylated	Tumor suppressor	Cervical cancer, Lung adenocarcinoma, Breast cancer, Oropharyngeal (oral) cancer, NSCLC	TGF-β1, BMF, Cyclin D1, JAG1	[46,47,48,49,50,51]
hsa-miR-34C	Downregulated	Hyper- methylated	Tumor suppressor	Nasopharyngeal carcinoma, Prostate cancer	MET	[52,53]
hsa-miR-34b/c	Downregulated	Hypermethylated	Tumor suppressor	CRC	-	[54]
hsa-miR-762	Upregulated	-	Tumor promoter	Breast cancer	IRF7	[55]
hsa-miR-9-3	Downregulated	Hypermethylated	Tumor suppressor	Hodgkin’s lymphoma, Gastric cancer	ITGB1	[56,57]
**Hypomethylated miRs Found in Our Study**	**miR Expression in Cancer**	**Regulation**	**Role**	**Cancer Types**	**Target Genes**	**References**
hsa-miR-1204	Upregulated	-	Tumor promoter	Breast cancer, Glioblastoma	VDR, CREB-1	[58,59]
hsa-miR-17	Upregulated	-	Tumor promoter	CRC	-	[60]
hsa-miR-18A	Upregulated	-	Tumor promoter	Prostate cancer, Breast cancers, Osteosarcoma, Nasopharyngeal carcinoma, CRC	STK4, IRF2, Dicer1	[14,61,62,63,64]
hsa-miR-19A	Upregulated	-	Tumor promoter	CRC, Gastric cancer, HCC	TIA1, MXD1, PTEN	[14,65,66,67,68]
hsa-miR-19B1	Upregulated	-	Tumor promoter	Gastric cancer	MXD1	[67]
hsa-miR-20A	Upregulated	-	Tumor promoter	CRC	WTX	[69]
hsa-miR-548F5	-	Hyper- methylated	-	Schwannomas	-	[70]

Abbreviations: B-cell lymphoma 2 (BCL2), BCL2-like 2 (BCL2L2), Bcl-2-modifying factor (BMF), CAMP responsive element binding protein 1 (CREB-1), Chemokine (C-C motif) ligand-20 (CCL20), CRK-like proto-oncogene, adaptor protein (CRKL), DNA methyl-transferase (DNMT3B), Enoyl coenzyme A hydratase short-chain 1 mitochondrial (ECHS1), Hepatocellular carcinoma (HCC), Histone-lysine N-methyltransferase (EZH2), Insulin-like growth factor-binding protein 7 (IGFBP7), Integrin Subunit Beta 1 (ITGB1), integrin αV endothelin receptor type B (EDNRB), Interferon regulatory factor 7 (IRF7), Interferon regulatory factor (IRF)2, Jagged1 (JAG1), Kruppel-like factor 12 (KLF12), Lysine demethylase (KDM4A), Lysine-specific demethylase 1 (LSD1), Lysine-specific histone demethylase 1A (LSD1), Max dimerization protein 1 (MXD1), MET proto-oncogene, receptor tyrosine kinase (MET), Non-small cell lung cancer (NSCLC), Phosphatase and tensin homolog (PTEN), Serine/threonine-protein kinase 4 (STK4), Signal transducer and activator of transcription 3 (STAT3), Specificity protein 1 (Sp1), SRY-related HMG-box (SOX4), T-cell intracellular antigen 1 (TIA1), Transcription factor 4 (TCF4), Transforming growth factor beta 1 (TGF-β1), Vitamin D receptor (VDR), Wilms tumor gene on the X chromosome (WTX).

## Data Availability

The array data have been deposited in the NCBI Gene Expression Omnibus and can be accessed using the GEO Series accession number GSE184494 (https://www.ncbi.nlm.nih.gov/geo/query/acc.cgi?acc=GSE184494, accessed on 20 September 2021).

## Supplementary Materials

The following are available online at https://www.mdpi.com/article/10.3390/cancers13235951/s1, Table S1: Clinical pathological characteristics of the tumour samples used for the methylation analysis. pT, pN, M stipulates the tumour node metastasis classification of the UICC, G reflects the tumour’s grade of differentiation. All tumours were microsatellite stable.
