# Supplementary material for: Changes in Methylation across Structural and MicroRNA Genes Relevant for Progression and Metastasis in Colorectal Cancer
Source: Cancers (Basel). 2021 Nov 26;13(23):5951. doi: 10.3390/cancers13235951 (PMC8656781; doi:10.3390/cancers13235951)
Supplement: Supplementary file 1 [file cancers-13-05951-s001.zip › cancers-1480973-supplementary.pdf]

cancers

MDPI

Table S1. Clinical pathological characteristics of the tumour samples used for the methylation analysis. pT, pN, M stipulates the tumour node metastasis classification of the UICC, G reflects the tumour's grade of differentiation. All tumours were microsatellite stable.

Samples No	Gender	Tumor type	Stage	pT	pN	G	M
1	Female	Colon	2	3	0	2	0
2	Male	Colon	4	4	2	2	1
3	Male	Colon	2	3	0	2	0
4	Male	Colon	2	3	0	2	0
5	Male	Colon	2	3	0	2	0
6	Female	Colon	3	3	0	2	MX
7	Male	Rectum/Sigma	1	pT2/ Sig.pT1	pN0 /Sig.pN0	G2/ Sig.G1/2	M0
8	Female	Rectum	3	4	1	2	M0
9	Male	Colon	3	3	2	3	M0
10	Female	Sigma	4	3	2	3	1
11	Male	Rectum	1	2	0	2	0
12	Male	Rectum	3	2	1	3	0
13	Female	Rectum	4	3	2	2	1
14	Male	Rectum	2	4	0	2	0
15	Male	Sigma	3	3	1	2	0
16	Male	Colon/Sigma	3	4	2	3	0
17	Female	Colon	4	3	2	3	1
18	Female	Recto-sigmoid	2	3	0	2	0
19	Male	Colon	1	2	0	2	0
20	Male	Colon	4	1	1	2	1
21	Male	Rectum	2	3	0	1	MX
22	Female	Colon	3	3	1	2	MX
23	Male	Rectum	3	2	2	2	MX
24	Female	Sigma	2	3	0	2	MX
